# L’évaluation d'un programme d’éducation thérapeutique chez le patient diabétique dans un Centre Hospitalier Universitaire marocain: résultats préliminaires d'une enquête pilote

**DOI:** 10.11604/pamj.2014.18.258.3054

**Published:** 2014-07-27

**Authors:** Sana Doubi, Hanan El Ouahabi, Otmane Dakkar, Farida Ajdi

**Affiliations:** 1 Service d'Endocrinologie, Diabétologie et Maladies Métaboliques, CHU Hassan II, Fès, Maroc; 2 Université Sidi Mohamed Benabdellah, Fès, Maroc

**Keywords:** Patient diabétique, éducation thérapeutique, prévention, diabetic patient, therapeutic education, prevention

## Abstract

L’éducation thérapeutique est fondamentale. Elle n'a pas uniquement pour effet la préservation du capital de santé par le meilleur contrôle métabolique, favorisé par la responsabilisation et l'autonomie. En effet, en se soignant mieux, le diabétique peut améliorer sa qualité de vie, et en retirer un bénéfice qui va au-delà de la satisfaction de préserver son avenir. L'objectif principal de cette étude était de déterminer le nombre des patients diabétiques ayant bénéficié d'une éducation thérapeutique, la relation entre cette éducation et les paramètres sociodémographiques de la population étudiée et clinico-biologiques liées à la maladie. Nous avions réalisé une étude transversale, étalée sur 3 mois, intéressant 100 patients diabétiques suivis au service d'Endocrinologie au CHU Hassan II Fès. L’âge moyen des patients était de 50,01 ans +/-16,44; 60% des patients étaient des femmes, la majorité des patients étaient des diabétiques de type2 (81 malades). La majorité des patients avaient un niveau d'instruction bas (41% non scolarisés), 69% appartenaient au milieu urbain. Alors que 62 malades n'avaient pas de couverture sociale. L’éducation thérapeutique avait compris différents thèmes: 91% des patients avaient bénéficié d'une éducation sur les mesures hygiéno-diététiques (néanmoins 34% ne les respectaient pas); 98% sur l'auto mesure glycémique (cependant 28% des patients ne surveillaient pas leurs glycémies régulièrement); 59% sur l'intérêt de HbA1c; 79% sur les complications du diabète et la majorité des patients avaient bénéficié d'une éducation sur le traitement antidiabétique (80%). L'analyse des données a mis en évidence une relation statiquement significative entre le niveau socioéconomique, le niveau d’éducation, le milieu de vie et l'application des différents volés de l’éducation thérapeutique: les mesures hygiéno-diététiques, le suivi médical et l'observance du traitement. L'intérêt de l’éducation thérapeutique dans la prise en charge des patients diabétiques est certain, un grand effort est nécessaire pour agir sur les facteurs l'entravant: l'analphabétisme, le manque de couverture sociale et surtout le bas niveau socioéconomique.

## Introduction

Les maladies chroniques sont des affections de longue durée, stables ou évolutives, qui ne peuvent être guéries définitivement mais dont, en règle générale, la progression ou du moins les symptômes peuvent être contrôlés. Elles nécessitent un suivi médical et un changement dans les habitudes de vie. Dans ce contexte, l’éducation thérapeutique constitue un élément de base dans la prise en charge. Dans ce travail, on avait tenté d'explorer cette dimension de l’éducation thérapeutique des patients diabétiques, à travers d'une part l’évaluation de l’état de connaissance du patient diabétique sur sa maladie, d'autre part par l’étude des différents facteurs ayant un impact direct ou indirect sur cette éducation.

## Méthodes

Il s'agit d'une étude transversale étalée sur 3 mois évaluant le système d’éducation thérapeutique instauré depuis un an chez les patients diabétiques consultant au service d'Endocrinologie Diabétologie du Centre Hospitalier Universitaire Hassan II de Fès au Maroc.

**Les critères d'inclusion:** Tous les diabétiques de type 1 et type 2 suivis au service ayant bénéficié on non de l’éducation thérapeutique, avec ou sans complications dégénératives.

**Les critères d'exclusion:** Diabète récent < 12 mois, diabètes secondaires.

**Modalités du recueil des données:** Nous avions effectué notre travail grâce à des fiches d'exploitation préalablement établies, comportant les éléments en rapport avec la maladie ainsi que l’éducation thérapeutique et remplie par le même médecin.

**Variables étudiées:** Chez tous les patients, nous avions étudié les variables suivantes: **Sociodémographiques:** l’âge, le sexe, l'origine géographique, le niveau d'instruction, le niveau socio-économique, la couverture sociale, la langue parlée; **Cliniques:** le type du diabète, l'ancienneté du diabète, l'hérédité diabétique dans la famille, les facteurs de risque cardiovasculaires, les aspects thérapeutiques et les complications dégénératives du diabète; **Biologiques:** le taux d'hémoglobine glyquée (HbA1c), les paramètres lipidiques, la clairance de la créatinine, l'examen cytobactériologique des urines, la microalbuminurie de 24h, et dans certains cas la protéinurie des 24 heures; **L’éducation thérapeutique du patient:** Nous avions étudié les différents aspects de cette éducation, incluant le respect des mesures hygiéno-diététiques, l'activité physique, le suivi médical, l'auto-surveillance glycémique ainsi que les différents thèmes éducatifs dont a bénéficié le patient. Les données ont été saisies sur Excel et analysées par la version 17 du logiciel SPSS.

## Résultats

### Population étudiée

Le nombre de patients recrutés était de 100 patients. Les extrêmes d’âge des patients oscillaient de 16 à 79 ans avec une moyenne d’âge de 50,01 ans +/-16,44, et un sexe ratio F/H 1,5. La majorité des patients diabétiques étaient de type2: (81%), la duré d’évolution du diabète était en moyenne de 7,8 +/-9,5 ans. avec des extrêmes allant de 1 ans à 34 ans Concernant les caractéristiques sociodémographiques, 41% des patients n’étaient pas scolarisés. 62% avaient un bas niveau socio-économique, alors que 69 malades appartenaient au milieu urbain.

Les facteurs de risque associés au diabète ainsi que les complications dégénératives macro et micro-angiopathiques sont représentés dans le Tableau 1. Chez la population étudiée, seulement 28% des diabétiques avaient un équilibre strict avec une HbA1c≤ 7%, 17% avaient un équilibre moyen HbA1c ≤ 7,9%, plus que la moitié des patients (55%) avaient une HbA1c ‘ 8%. La majorité des diabétiques était sous traitement médical (antidiabétiques oraux, insuline ou association). Plus de la moitié des patients (56%) avaient une observance thérapeutique optimale (aucun oubli de prise médicamenteuse par mois), 27% une mauvaise observance (au moins 4 oublis de prise médicamenteuse par mois)

**Tableau 1:** Pourcentage des facteurs de risque cardiovasculaire et complications dégénératives: (nombre de patients: 100)

	%
HTA	35
Dyslipidémie	23
Tabagisme	16
Complications dégénératives	55
Rétinopathie	19
Microalbuminurie positive	14
Insuffisance rénale	9
Débutante	9
Modérée	4
Sévère	1
Syndrome coronaire	9
AOMI	4
AVC	5

### L’évaluation de l’éducation thérapeutique

Des 100 cas recrutés, 91% avaient bénéficié d'une éducation en ce qui concerne le diabète tout thème inclus. 82% des malades avaient eu un suivi médical pendant ces 12 derniers mois alors que 18% n’étaient pas suivis. Ce suivi a été assuré par un spécialiste chez 34% des patients, 24% étaient suivis par un généraliste et 42% avaient un suivi par les deux.

Plus de la moitié des patients (59%) n'avaient pas bénéficié d'une éducation sur l'intérêt de HbA1c ou ne comprenaient pas sa signification exact et ceci est du à plusieurs facteurs essentiellement l'analphabétisme, alors que 21% des malades ne savaient pas où avaient une connaissance limitée sur les complications aigues et chronique du diabète. Chez 39% des malades, il n'y avait aucune connaissance sur les effets indésirables et les interactions médicamenteuses.

**Figure 1:**
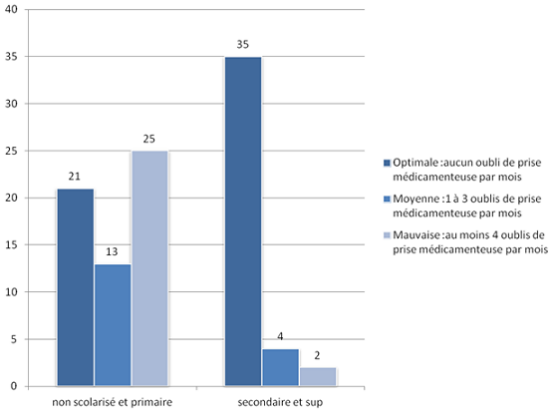
Répartition des groupes selon le niveau scolaire

**Figure 2:**
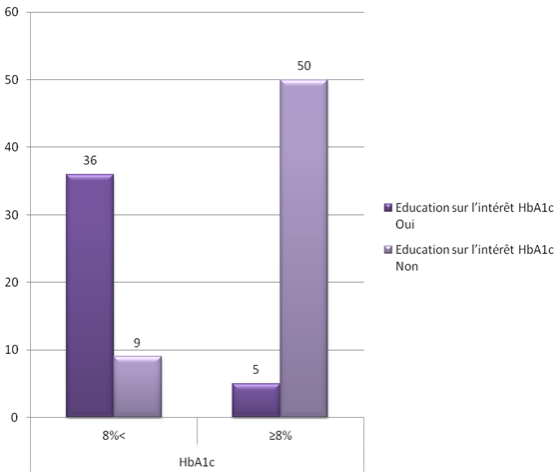
Equilibre glycémique chez les deux groupes de diabétiques

**Figure 3:**
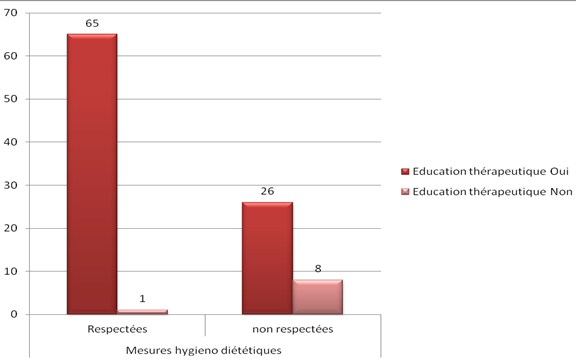
Respect des MHD chez les deux groups

**Figure 4:**
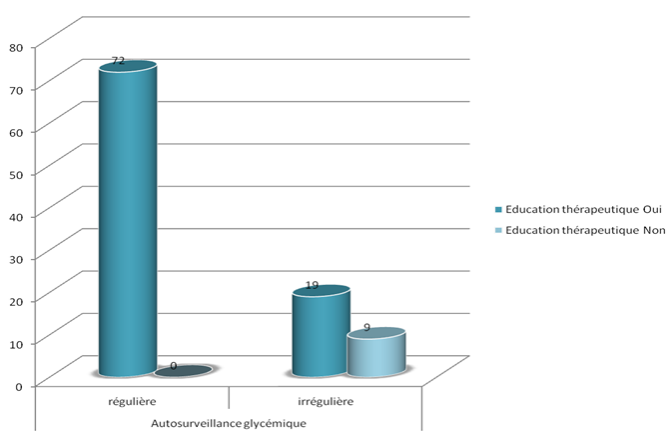
Auto-surveillance glycémique chez les deux groupes

Presque tous les patients (98%) avaient bénéficié d'une éducation sur l'auto-surveillance glycémique. L’évaluation de cette éducation avait montré que 66% des patients respectaient les mesures hygiéno-diététiques, alors que 34% ne le faisaient pas pour des raisons différentes: (difficulté d'adaptation à ces mesures, ou manque de volonté). Nous avions étudié les paramètres liés au terrain et la maladie et leur relation avec l'adhésion aux instructions de l’éducation thérapeutique. Concernant le niveau socioéconomique, la relation était statistiquement significative en ce qui concerne le respect ou non des mesures hygiéno-diététiques (MHD) (p = 0.002), le suivi médical (p = 0,09), et l'observance thérapeutique (p= 0,03). Aussi, il y avait une relation statistiquement significative entre le niveau scolaire et le respect ou non des MHD (p= 0,001), le suivi médical (p= 0,01), et l'observance (p= 0,001) (Figure 1). La relation entre l’équilibre glycémique et les patients qui avaient bénéficié d'une éducation sur l'intérêt de HbA1c était significative sur le plan statistique (p = 0.001)(Figure 2). Aussi, les patients ayant bénéficié d'une éducation thérapeutique respectaient mieux les MHD que les patients non éduqués (p= 0,001) (Figure 3), et pratiquaient l'auto-surveillance glycémique de façon régulière (p= 0,001) (Figure 4). Par ailleurs, il n'a pas été noté de lien significatif entre les complications du diabète et l’éducation thérapeutique.

## Discussion

L’éducation thérapeutique du patient diabétique est fondamentale. Elle est destinée à aider le patient et sa famille et/ou son entourage à comprendre la maladie et les traitements, collaborer aux soins, prendre en charge son état de santé et conserver et/ou améliorer sa qualité de vie [1]. Le terme générique d’éducation du patient recouvre 3 types d'activités [2]: L’éducation pour la santé qui concerne tant la maladie que les comportements de santé et les modes de vie du patient; L’éducation du patient à sa maladie qui concerne les comportements de santé liés au traitement, à la prévention des complications et rechutes et autres comportements non médicaux liés à l'existence de cette maladie; L’éducation thérapeutique du patient qui touche à la partie de l’éducation directement liée au traitement (curatif ou préventif). Ce volet de l’éducation fait donc partie de la fonction soignante et du traitement.

Dans notre étude, Le niveau d’étude semble avoir une influence très significative (P < 10^−3^), le même résultat est trouvé dans l’étude QUODIEM [3]; l’étude américaine de J. Todd Coffey et al [4]. Nos résultats pourraient alors s'expliquer par le bais d'une meilleure compréhension de la maladie chez les patients instruits. Dans notre série la relation entre le niveau socio économique ainsi que l'observance est très marquée, plus de 60% de la population étudiée à un bas niveau socioéconomique, dans de nombreux pays en développement, la pauvreté représente une barrière majeure pour la prise en charge de toutes les maladies en général, et celle des affections chroniques plus particulièrement [5]

### Influence de l’éducation sur l’équilibre glycémique

Plusieurs études montrent que l’éducation à l'autogestion du diabète par le patient est efficace sur le taux d'HbA1c: Dans une méta-analyse réalisée en 2002 [6] sur 31 études, Norris SL et al. montrent que l’éducation à l'autogestion du diabète de type 2 améliore immédiatement pendant le suivi les taux d'HbA1c. Dans une autre méta-analyse réalisée en 2004 [7], Warsi A et al. montrent que les patients diabétiques impliqués dans des programmes d’éducation à l'autogestion ont une diminution de leur HbA1c. Dans notre série l'auto-surveillance glycémique régulière est plus marquée, et l’équilibre glycémique est meilleur chez les patients éduqués la relation entre ces deux paramètres est significative sur le plan statistique (p = 0,001).

### Influence de l’éducation sur la prévention des complications

Les études de cohortes [8], les études prospectives interventionnelles randomisées (notamment DCCT pour le diabète de type 1 [9] et UKPDS pour le diabète de type 2 [10]) ont permis de montrer qu'une amélioration de l’équilibre glycémique et le contrôle des autres facteurs de risque cardiovasculaires diminuaient l'incidence des complications. Réduire les facteurs de risque implique la compréhension, la recherche et le maintien de plusieurs services de soins de santé préventifs sur une base périodique, tels que les examens ophtalmiques annuels, le suivi médical de routine et les examens dentaires. Il existe de nombreuses preuves démontrant l'importance et l'efficacité des thérapies nutritionnelles médicales sur le diabète [11, 12]. La prescription nutritionnelle peut entraîner une baisse de 1% à 2% du taux d'HbA1c [13], une diminution de la pression artérielle ainsi qu'une perte de poids significative par semaine.

## Conclusion

L'intérêt de l’éducation thérapeutique dans la prise en charge des patients diabétiques est certain, un grand effort est nécessaire pour agir sur les facteurs entravant: l'analphabétisme, le manque de couverture sociale et surtout le bas niveau socioéconomique. La réussite donc d'un programme d’éducation thérapeutique est intimement liée à son adaptation au contexte où il se déroule.
